# Blood Pressure and Arterial Stiffness in Kenyan Adolescents With α^+^Thalassemia

**DOI:** 10.1161/JAHA.117.005613

**Published:** 2017-04-05

**Authors:** Anthony O. Etyang, Christopher Khayeka‐Wandabwa, Sailoki Kapesa, Esther Muthumbi, Emily Odipo, Marylene Wamukoya, Nicholas Ngomi, Tilahun Haregu, Catherine Kyobutungi, Metrine Tendwa, Johnstone Makale, Alex Macharia, J. Kennedy Cruickshank, Liam Smeeth, J. Anthony G. Scott, Thomas N. Williams

**Affiliations:** ^1^ KEMRI‐Wellcome Trust Research Program Kilifi Kenya; ^2^ London School of Hygiene and Tropical Medicine London United Kingdom; ^3^ African Population and Health Research Centre Nairobi Kenya; ^4^ King's College London United Kingdom; ^5^ Imperial College London United Kingdom

**Keywords:** adolescence, ambulatory blood pressure monitoring, α^+^thalassemia, Endothelium/Vascular Type/Nitric Oxide, Vascular Biology, Genetics, High Blood Pressure

## Abstract

**Background:**

Recent studies have discovered that α‐globin is expressed in blood vessel walls where it plays a role in regulating vascular tone. We tested the hypothesis that blood pressure (BP) might differ between normal individuals and those with α^+^thalassemia, in whom the production of α‐globin is reduced.

**Methods and Results:**

The study was conducted in Nairobi, Kenya, among 938 adolescents aged 11 to 17 years. Twenty‐four‐hour ambulatory BP monitoring and arterial stiffness measurements were performed using an arteriograph device. We genotyped for α^+^thalassemia by polymerase chain reaction. Complete data for analysis were available for 623 subjects; 223 (36%) were heterozygous (−α/αα) and 47 (8%) were homozygous (−α/−α) for α^+^thalassemia whereas the remaining 353 (55%) were normal (αα/αα). Mean 24‐hour systolic BP ±SD was 118±12 mm Hg in αα/αα, 117±11 mm Hg in −α/αα, and 118±11 mm Hg in −α/−α subjects, respectively. Mean 24‐hour diastolic BP ±SD in these groups was 64±8, 63±7, and 65±8 mm Hg, respectively. Mean pulse wave velocity (PWV)±SD was 7±0.8, 7±0.8, and 7±0.7 ms^−1^, respectively. No differences were observed in PWV and any of the 24‐hour ambulatory BP monitoring‐derived measures between those with and without α^+^thalassemia.

**Conclusions:**

These data suggest that the presence of α^+^thalassemia does not affect BP and/or arterial stiffness in Kenyan adolescents.

## Introduction

The thalassemias, in which there is disordered or absent production of the α‐ or β‐globin chains that make up normal hemoglobin, are the most common monogenic disorders of humans.^1^ The geographical distribution of α^+^thalassemia, in which there is deletion of 1 or more of the *HBA* genes that encode α‐globin (Hbα) production, closely mirrors that of malaria transmission,^2^ and it has been demonstrated that these deletions confer protection against both severe and nonsevere malaria.^2^, ^3^, ^4^, ^5^

Although it has long been believed that Hbα expression is limited to red blood cells, it has recently been demonstrated that Hbα is also expressed in mouse endothelial cells where it plays a role in nitric oxide (NO) signaling, influencing vascular smooth muscle tone in resistance arteries.^6^, ^7^ A macromolecular complex formed by Hbα and endothelial nitric oxide synthase (eNOS), regulates NO signaling at myoendothelial junctions.^8^ Disruption of this complex lowers blood pressure (BP) in both normotensive and hypertensive mice.^8^ It has also been shown that resistance arteries from mice lacking 2 of the 4 α‐globin genes (−α_2_/−α_2_) have reduced contractility posttreatment with the vasoconstrictor, phenylephrine.^9^ Individuals with α^+^thalassemia have been shown to have higher microvasculature tortuosity.^10^ From the foregoing, it could be expected that individuals with α^+^thalassemia might have lower BP compared with those with normal hemoglobin. However, the few studies conducted in humans have yielded inconsistent results. Whereas 1 review^11^ suggested that α^+^thalassemic individuals have moderate hypotension, other investigators have found elevated BPs in subjects with this condition.^12^, ^13^ These studies were limited by small sample sizes and the failure to use 24‐hour ambulatory blood pressure monitoring (ABPM) to measure BP. It is known that 1‐off office/clinic BP measurements can be influenced by a variety of environmental and psychological factors,^14^ limitations that are overcome by use of ABPM, which is considered the reference standard for BP measurement.^14^, ^15^

If arterial stiffness and/or BP are influenced by Hbα genotype, this would be an important step that could aid the development of compounds either mimicking or antagonizing Hbα as potential therapies for hypertension. In the current study, we have tested the hypothesis that 24‐hour BP and arterial stiffness is different in subjects with α^+^thalassemia than in healthy individuals.

## Methods

This population‐based study was a cross‐sectional sample of residents of the Nairobi Urban Health and Demographic Surveillance System (NUHDSS)^16^ in Kenya and was conducted between December 2015 and June 2016. Nairobi, the capital city of Kenya, was chosen for this study for 2 reasons. First, Nairobi is located at a high altitude (1800 m above sea level), and there is no evidence of malaria transmission.^17^ This made it possible to study the effect of α^+^thalassemia on BP unconfounded by the presence of malaria, which could potentially influence BP^18^ and which α^+^thalassemia protects against. Second, the population of Nairobi is composed of ethnic groups originating from all parts of the country, including those whose ancestral lands were endemic for malaria (eg, Luhya, Luo, Teso, and Mijikenda), in whom the frequency of α^+^thalassemia is significantly higher.^2^ In order to increase our efficiency in recruiting participants with α^+^thalassemia, we limited our recruitment to those who identified themselves as genetically derived from any of these ethnic groups.

The NUHDSS conducts population‐wide censuses within the study area 4 times each year.^16^ Using NUHDSS data, we selected all children currently aged 11 to 17 years who had a continuous record of residence within the study area since birth. Continuous residency was a requirement in order to minimize potential exposure to malaria as a result of migration. Trained staff visited all subjects who had been selected to participate in the study at their homes. Parents of the children were then asked to bring them to the nearer of 2 study clinics within the area to undergo study procedures. Up to 3 attempts were made at finding a selected subject before concluding that they could not be found. Subjects who failed to come to the clinic within 3 months of being invited were considered to have declined to participate in the study.

Recruited subjects first underwent an interview where they answered questions about their past medical history and their socioeconomic status based on the multidimensional poverty index.^19^ Weight and height were measured using a validated SECA 874 weighing machine and a portable stadiometer (SECA 213), respectively. Mid‐upper‐arm circumference (MUAC) was measured in a standardized manner using TALC MUAC tapes. We then took a screening BP measurement using a validated Omron M10‐IT sphygmomanometer. An appropriately sized cuff was placed on the nondominant arm after the subject had been seated for at least 5 minutes. Three BP measurements were taken over a 5‐minute period, and the mean of the last 2 measurements was recorded as the screening BP value. All participants were subsequently fitted with a validated Arteriograph24 device for 24‐hour ABPM as well as pulse wave velocity (PWV) determination.^20^ These devices were programmed to take measurements every 20 minutes from 6:00 am to 10:00 pm and every 40 minutes from 10:00 pm to 6:00 am.

Because there are no published criteria for acceptable ABPM data in children, we used guidelines for completeness of ABPM data in adults from the International Database of Ambulatory blood pressure in relation to Cardiovascular Outcomes (IDACO) study.^21^ Specifically, ABPM data were considered of acceptable quality if they included a minimum 10 daytime and minimum 5 nighttime readings, where daytime was defined as 10:00 am to 10:00 pm and nighttime as 12:00 am to 6:00 am.^21^ The same time periods were used to determine average daytime and nighttime BPs and evaluate dipping status. Time weighting was applied in calculating average BP values for all time periods.^22^

We defined screen positives for hypertension as individuals whose mean of the last 2 clinic BP measurements was above the 95th percentile for their age, sex, and height.^15^ Confirmed hypertensives were those whose 24 hours systolic (SBP) and/or diastolic BP (DBP) averages, respectively, were above the 95th percentile for their sex, age, and height.^15^

We categorized all subjects who were not on antihypertensive medication using the combination of clinic BP measurements and ABPM into 4 categories: sustained hypertensives (screen positive and confirmed hypertensive on ABPM); white coat hypertensives (screen positive, not confirmed hypertensive on ABPM); masked hypertensives (screen negative, confirmed hypertensive on ABPM); or normotensives (screen negative, not confirmed hypertensive on ABPM).^23^

Dipping status was defined using ABPM data only, using day and night periods as defined above. Subjects were classified using the following 4 categories, based on the night/day ratio of mean SBP and/or DBP: rising or absence of dipping (ratio ≥1.0); mild dipping (0.9< ratio ≤1.0); dipping (0.8< ratio ≤0.9); and extreme dipping (ratio ≤0.8).^24^

### Laboratory Procedures

We collected 10 mL of blood from participants for full blood count, determination of α^+^thalassemia genotype, and serum electrolytes. After performing automated full blood counts using an ACT 5 machine, whole blood samples were frozen at −80°C and then transported to the KEMRI‐Wellcome Trust Research Programme laboratories in Kilifi, Kenya, for genotyping. DNA was extracted retrospectively from frozen samples by use of Qiagen DNA blood mini‐kits (Qiagen, Crawley, UK) and typed for the common African −3.7‐kb *HBA* deletion by polymerase chain reaction.^25^

Serum and urine samples collected from participants were frozen at −80°C within 4 hours of collection and later transported to Kilifi, Kenya, for subsequent analysis. We determined sodium and potassium, urea, and creatinine levels in these samples using ion electrophoresis and the Jaffe method, respectively.^26^ We additionally determined albumin levels in urine samples by immunoturbidometry using a Quantex microalbumin kit.

Estimated glomerular filtration rate (eGFR) was calculated using the Schwarz formula.^27^

### Statistical Analysis

Based on an expected minimum prevalence for heterozygous α^+^thalassemia (−α/αα) of 20% in the ethnic groups we were studying, an SBP SD of 15 mm Hg, and 30% attrition because of poor‐quality ABPM data, we estimated that a total of 472 participants would provide 80% power to detect one third of an SD (5 mm Hg) difference in 24‐hour SBP between −α/αα and αα/αα individuals.

Summary statistics that were computed included means, medians, and proportions as appropriate. We used the Student *t* test to separately compare continuous variables in −α/αα and −α/−α to αα/αα individuals. The chi‐squared test was used to compare categorical variables. We conducted multiple regression analyses to determine whether inclusion of α^+^thalassemia genotype predicted 24‐hour SBP and/or DBP. Age, sex, BMI, eGFR, and PWV, which have all been previously associated with BP, were included as covariates in the base model. To determine whether α^+^thalassemia genotype influenced 24‐hour BP, we added it to the base model and used the likelihood ratio test to determine whether it improved the fit. We additionally tested for interaction with the sickle cell trait, because it has previously been associated with cardiovascular and renal events.^28^, ^29^, ^30^, ^31^ All analyses were conducted using Stata software (version 12; StataCorp LP, College Station, TX).

The Kenya Medical Research Institute's Ethical Review Committee approved the study. Written informed consent was obtained from parents of study participants. Participating children also provided written assent.

## Results

**Figure 1:**
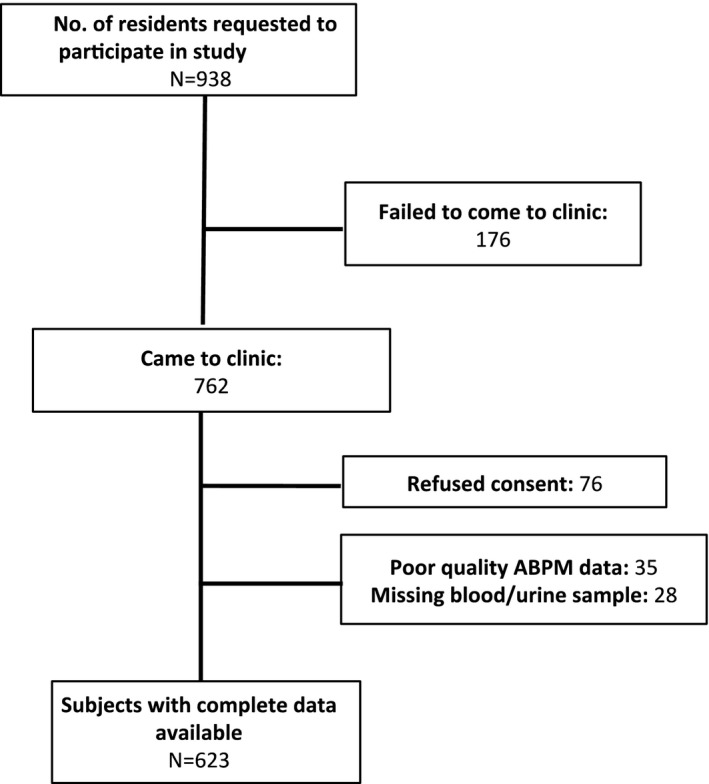
Study flow chart.

Of the 938 subjects invited to participate in the study, 686 completed enrollment (Figure 1). None of the participants were previously aware of their α^+^thalassemia status. The 252 adolescents that were not recruited into the study were 0.6 years (95% CI, 0.3–0.9) older than study participants, but with a similar sex distribution (53% female) to those that participated in the study. Data on α^+^thalassemia genotype were available for 664 (97%) participants. Two hundred forty‐six (37%) were heterozygous (−α/αα) and 49 (7%) were homozygous (−α/−α) for α^+^thalassemia whereas the remaining 369 (56%) subjects were normal (αα/αα). One hundred three (15.5%) of the adolescents were carriers of the sickle cell trait, distributed equally among the α^+^thalassemia genotypic groups (14%, 17%, and 16% in αα/αα, −α/αα, and −α/−α subjects, respectively; *P*=0.652). After excluding those with poor‐quality ABPM data, 623 (94%) subjects provided quality data for the analysis (Figure 1). A slightly lower proportion of −α/αα participants had complete ABPM data (91%) compared with αα/αα (95%) and −α/−α subjects (97%). Mean clinic BP±SD among all participants was 98±11 mm Hg SBP and 64±8 mm Hg DBP. Mean 24‐hour BP±SD for all participants was 117±12 mm Hg SBP and 64±8 mm Hg DBP. Mean 24‐hour pulse wave velocity (PWV)±SD was 7±0.8 ms^−1^. The study had >98% power to detect one third of an SD difference in either SBP or DBP (4 and 2.7 mm Hg, respectively) between αα/αα individuals and those with −α/αα and a 0.3 ms^−1^ (one third of an SD) difference in PWV between αα/αα individuals and those with −α/αα. The study had >90% power to detect differences equivalent to 0.5 SDs in BP and PWV between αα/αα and −α/−α individuals.

Table 1 displays the characteristics of study participants according to α^+^thalassemia genotype. As expected, hemoglobin concentrations were significantly lower in −α/−α than in −α/αα or αα/αα subjects. BMI was lower in −α/αα than in αα/αα individuals (18.2 versus 19.2; *P*=0.0004) whereas MUAC was significantly smaller in −α/αα compared with αα/αα individuals. There were no statistically significant differences in prevalence of masked hypertension, white coat hypertension, or in the pattern of nondipping BP by α^+^thalassemia genotype. PWV was also similar in all 3 groups.

**Table 1:** Characteristics of Study Participants (N=623)

Characteristic	Normal (αα/αα) N=353	Heterozygous (−α/αα) N=223	Homozygous (−α/−α) N=47	*P* Value*	*P* Value†
Age, y	13.4 (2.2)	13.0 (2.2)	13.4 (2.4)	0.0289	0.9
Female, N (%)	187 (53)	132 (59)	28 (58)	0.3	0.3
BMI, kg/m^2^	19.2 (3.2)	18.3 (2.6)	19.2 (3.6)	0.0004	0.9
MUAC, cm	23.7 (3.9)	22.7 (3.1)	23.2 (4)	0.0007	0.4
Hemoglobin, mg/dL	13.5 (1.5)	13.1 (1.4)	12.2 (1.6)	0.0004	<0.0001
Mean cell volume, fL	84 (5)	79 (5)	70 (5)	<0.0001	<0.0001
Mean corpuscular hemoglobin concentration, g/dL	32 (2)	31 (2)	31 (2)	0.0003	<0.0001
Socioeconomic status (MDPI score)	2.0 (1.2)	2.3 (1.4)	2.4 (1.1)	0.0126	0.3
24‐hour SBP, mm Hg	118 (12)	117 (11)	118 (11)	0.1	1.0
24‐hour DBP, mm Hg	64 (8)	63 (7)	65 (8)	0.1	0.6
24‐hour pulse wave velocity, ms^−1^	7.0 (0.8)	7.0 (0.8)	7.0 (0.7)	0.2	0.5
Systolic morning BP surge, mm Hg	9 (12)	8 (12)	11 (10)	0. 6	0.2
Augmentation index, %	17 (6)	17 (6)	16 (5)	0.8	0.4
White coat hypertension, N (%)	15 (4)	8 (4)	3 (6)	0.2	0.4
Masked hypertension, N (%)	25 (7)	21 (9)	7 (15)	1.0	0.073
Nondipping BP pattern	24 (7)	7 (3)	2 (4)	0.1	0.9
eGFR, mL/min per 1.73 m^2^	109 (15)	111 (14)	110 (13)	0.1	0.6
Log_10_UACr	0.4 (0.6)	0.3 (0.7)	0.2 (1)	0.4	0.1
Urine sodium, mmol/L	137 (82)	130 (53)	128 (51)	0.3	0.5
Urine potassium, mmol/L	48 (32)	46 (29)	42 (20)	0.4	0.2

Data are mean (SD), unless specified. BP indicates blood pressure; DBP, diastolic blood pressure; eGFR, estimated glomerular filtration rate; MDPI, multidimensional poverty index; MUAC, mid‐upper‐arm circumference; systolic blood pressure; UACr, urine albumin‐to‐creatinine ratio.

*P* values are for comparisons between normal and heterozygous* and normal and homozygous^†^.

**Figure 2:**
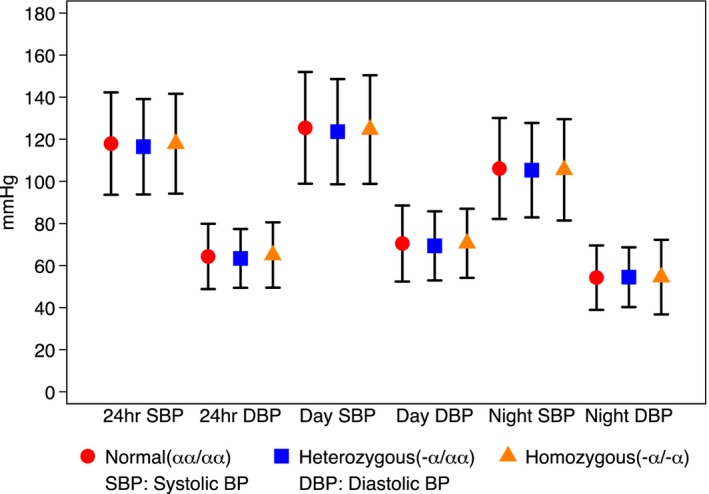
Twenty‐four hour ABPM measures by alpha thalassemia status. Data are mean and 95% CIs. ABPM indicates ambulatory blood pressure monitoring; BP, blood pressure.

Figure 2 displays mean 24‐hour, daytime, and nighttime BPs in study participants by α^+^thalassemia genotype. All measures were similar for all 3 groups.

The results of regression analyses are displayed in Table 2. Age, sex, BMI, eGFR, and PWV were all independent predictors of 24‐hour SBP whereas PWV was the only independent predictor of 24‐hour DBP. Twenty‐four‐hour BP values were not associated with α^+^thalassemia genotype in any of our regression models, and its inclusion in the final model did not improve the fit (likelihood ratio test, *P*=0.96 for SBP and *P*=0.75 for DBP). Adjustment for proxy markers of hemolysis (hemoglobin level, mean corpuscular volume, and mean corpuscular hemoglobin concentration) made no difference to the results, and neither did the inclusion of interaction terms for sex and sickle cell trait status.

**Table 2:** Regression Analyses Investigating Possible Effect of Thalassemia Status on 24‐Hour Systolic and Diastolic BP

	24‐Hour SBP		24‐Hour DBP	
	β, 95% CI	*P* Value	β, 95% CI	*P* Value
Age, y	0.6 (0.1–1.2)	0.021	0.03 (−0.3 to 0.4)	0.9
Male sex	2.6 (0.7–4.5)	0.009	0.2 (−1.1 to 1.4)	0.9
BMI, kg/m^2^	0.6 (0.2–1)	0.001	0.2 (−0.1 to 0.4)	0.2
PWV, ms^−1^	2.8 (1.6–4.1)	<0.001	2.7 (1.9–3.6)	<0.001
eGFR, mL/min per 1.73 m^2^	0.1 (0.03–0.2)	0.006	0.02 (−0.02 to 0.06)	0.4
α^+^thalassemia genotype	0.04 (−1.4 to 1.5)	1.0	0.1 (−0.8 to 1.1)	0.8

Likelihood ratio test for models including versus excluding α^+^thalassemia genotype *P*=1.0 for SBP and *P*=0.8 for DBP. BP indicates blood pressure; eGFR, estimated glomerular filtration rate; PWV, pulse wave velocity.

## Discussion

The α^+^thalassemias are the most common human monogenic disorders.^1^ Demonstration of altered BP in individuals with any of the mutations would be of immense importance, given that it would improve the understanding of BP regulation and aid the development of new drugs. In this detailed study of BP phenotypes and arterial stiffness among adolescents, we did not find any differences between those with and without α^+^thalassemia. Because the exposure measurement was a genetic trait acquired at conception and the participants were ascertained to have remained in the same malaria‐free environment since birth, we believe that this study suggests that a direct effect of α^+^thalassemia on BP and indices of arterial stiffness within the first 11 to 17 years of life is highly improbable.

On the face of it, our results do not align with findings from other studies that have suggested the possibility that expression of Hbα might affect BP.^6^, ^8^, ^11^ These studies were either done in vitro or in mouse models with very limited sample sizes (N=6).^8^ The review by Butcher et al^11^ that reported an association between α^+^thalassemia and moderate hypotension did not refer to a primary publication. It is possible that the lower BPs observed in subjects with α^+^thalassemia who are relatively protected from malaria could actually be a confirmation that malaria raises BP as we have previously hypothesized.^18^ An alternative explanation for similar BP despite the presence of Hbα deletions could be attributed to canalization, a phenomenon where individuals or organisms develop the same phenotype despite differences in their genetic makeup.^32^ Reddy et al^33^ have shown that infusion of HbH (levels of which are elevated in α^+^thalassemia) into rats results in elevation of BP as a result of HbH having higher affinity for NO than HbA.^34^ It is therefore possible that the BP‐lowering effect of α^+^thalassemia is cancelled out by the opposing effect of elevated levels of HbH. This would also suggest that recently developed molecules that mimic alpha globin^35^ may have reduced effectiveness in individuals with α^+^thalassemia. Additional studies are needed to fully understand these seemingly contrasting effects and generate a unified model incorporating both environmental conditions and genetic effects.

A major strength of this study was the use of ABPM, which is considered the reference standard for BP measurement in children.^15^ The study was well powered to detect very small differences in BP and PWV. Although it could be argued that PWV is predominantly a measure of large conduit arteries, which do not express Hb alpha, it integrates the interface between small arteries and resistance vessels—as, for instance, in diabetes mellitus where small vessel damage is as frequent as large.^36^ An additional strength of the study is that we used health and demographic surveillance system (HDSS) records that were prospectively collected in order to ascertain residence in a nonmalaria zone, there being no better method of doing this in sub‐Saharan Africa.

One limitation of this study was the limited age range of subjects recruited, necessitated by the fact that there were no long‐term residency records for older individuals. Most HDSSs in sub‐Saharan Africa were established in the late 1990s to early 2000s.^37^ Recruiting older individuals would have compromised data on residency status in childhood, the period when malaria risk is highest. Although BP differences are likely to be larger at older ages, it is known that differences in adult BP emerge in childhood^38^, ^39^ and that childhood BP levels are predictive of adult BP.^40^ The absence of even a small difference in carefully measured BP and arterial stiffness in our study of adolescents therefore suggests that it is very unlikely such differences would emerge in future.

A second limitation of the study is the fact that we did not measure levels of markers of hemolysis, such as HbH and haptoglobin, and other potential compensatory mechanisms, such as (decreased) eNOS or guanylyl cyclase expression, or increased catecholamine levels among study participants. This would have helped to either confirm or refute the possibility of canalization explaining the lack of an effect of α^+^thalassemia on BP levels. This could form the basis of future studies to better understand the seemingly contrasting findings of experimental and human studies.

It is also important to note that no studies have to date established whether alpha hemoglobin is expressed in endothelial cells of human subjects and whether the 3.7‐kb deletion, the most common defect causing α^+^thalassemia^1^ in humans, also results in reduced endothelial expression of alpha hemoglobin. Additional studies are required to determine whether there is endothelial expression of alpha hemoglobin in humans, whether the 3.7‐kb deletion results in reduced endothelial α globin expression, and whether other defects resulting in α^+^thalassemia present with the same vascular phenotype that we observed.

In summary, we have demonstrated that there are no differences in BP and arterial stiffness based on α^+^thalassemia genotype in Kenyan adolescents living within a non‐malaria‐endemic environment. Additional studies are required to explain the apparent contradictory results of experimental studies.

## Sources of Funding

Etyang, Smeeth, Williams, and Scott are funded by the Wellcome Trust (Fellowship Nos.: 103951/Z/14/Z, 098532, 091758, and 098504). The funders played no role in the preparation of this article.

## Disclosures

None.
